# Mechanical Tricuspid Valve Thrombosis and Pregnancy

**DOI:** 10.3390/life16060886

**Published:** 2026-05-25

**Authors:** Míriam Mayal Etreros, Susana Beltrán Martínez, Diana Domingo Valero, Carmen Padilla Prieto, Javier Valero Domínguez, Javier Monleón Sancho, Dolores Borrás Suñer, Beatriz Marcos Puig, Vicente José Diago Almela

**Affiliations:** 1 Obstetrics and Gynecology, Hospital Universitari i Politècnic La Fe, 46026 Valencia, Spain; padilla_carpri@gva.es (C.P.P.); monleon_javsan@gva.es (J.M.S.); mdosun_borras@gva.es (D.B.S.); marcos_bea@gva.es (B.M.P.); 2 Cardiology, Hospital Universitari i Politècnic La Fe, 46026 Valencia, Spain; beltran_susmar@gva.es (S.B.M.); domingo_dia@gva.es (D.D.V.)

**Keywords:** pregnancy, mechanical tricuspid valve, valve thrombosis, anticoagulation, ischemic cesarean section

## Abstract

Mechanical tricuspid valve thrombosis during pregnancy is a rare but extremely high-risk condition due to the thrombogenic nature of tricuspid prostheses and the hypercoagulable state of pregnancy, which complicates anticoagulation management. This article reports the case of a 39-year-old pregnant woman who developed thrombosis of a mechanical tricuspid valve after switching from warfarin to low-molecular-weight heparin. Owing to her hemodynamic stability, a conservative approach with intensified anticoagulation and close multidisciplinary monitoring was adopted, allowing continuation of the pregnancy without maternal clinical deterioration. Partial echocardiographic improvement was observed. Delivery was achieved by planned ischemic cesarean section to minimize hemodynamic stress, resulting in a stable preterm neonate. The patient recovered well and subsequently underwent elective replacement of the mechanical valve with a bioprosthesis postpartum. This case highlights the complexity of diagnosing and managing mechanical tricuspid valve thrombosis during pregnancy and emphasizes the importance of individualized, multidisciplinary care, as well as the selective use of ischemic cesarean section in extreme-risk scenarios.

## 1. Introduction

Pregnancy in women with mechanical heart valves (MHVs) represents a complex clinical scenario associated with a high risk of complications for both the mother and the fetus (mWHO 2.0 risk classification ≥ III) [1,2,3,4,5,6]. Thrombotic events occur in 9–24% of cases, while hemorrhagic complications arise in 20–30%, both carrying a considerable risk of morbidity and mortality [1,3,4,5,6,7,8].

Clinical guidelines recommend appropriate anticoagulation in patients with mechanical valves. Initial anticoagulation during pregnancy should be performed with unfractionated heparin (UFH) or low-molecular-weight heparin (LMWH); however, in some cases it ultimately becomes necessary to switch anticoagulation to warfarin in order to achieve the target level of anticoagulation, despite its administration being contraindicated during pregnancy [1,5,7,8,9,10,11,12]. Mechanical valve thrombosis (MVT) frequently occurs during transitions from Vitamin K antagonist (VKAs) to heparin, particularly in the first trimester, making individualized anticoagulation strategies essential [5,10,11,12,13].

Given the complex and often controversial nature of anticoagulation management in pregnancy [4,14], the publication of such cases is crucial to enrich the limited existing evidence and to guide personalized therapeutic strategies in these challenging situations [2,4,7,14].

In this article, we present a case of mechanical tricuspid valve thrombosis in a pregnant woman, highlighting the diagnostic and therapeutic challenges encountered and contributing to the understanding of this rare but highly severe condition [3,7].

## 2. Case Presentation

A 39-year-old woman, a former smoker, was followed by the Cardiology and Hematology Departments at La Fe University Hospital. She had a mechanical tricuspid valve prosthesis implanted due to congenital dysplasia of the native valve and presented for evaluation during her second pregnancy. Her first pregnancy had been electively terminated because gestation was contraindicated in the setting of cardiac decompensation. She had no other relevant medical history.

Regarding her cardiac history, she underwent initial valve replacement with a 25 mm St. Jude prosthesis in 1997, requiring reoperation in 2003 due to valve malfunction, at which time it was replaced with a 27 mm St. Jude prosthesis. Subsequently, she experienced several episodes of valve thrombosis treated with aspirin, acenocoumarol, and heparin. On two occasions, she required slow infusion fibrinolysis, as well as an episode of atrial flutter resolved by external cardioversion. Her most recent episode of prosthetic valve thrombosis occurred in 2021 during the transition from warfarin to heparin in the context of pregnancy.

During follow up in the cardiology clinic, the patient expressed a desire for pregnancy. The management plan included early notification of pregnancy to allow prompt transition to low-molecular-weight heparin (LMWH), targeting anti Xa levels between 1 and 1.2 U/mL, switching to warfarin in the second trimester, and reintroducing heparin at 34–36 weeks of gestation.

Baseline echocardiographic evaluations prior to the thrombotic episode showed a pressure half time of 120–130 ms and a mean gradient of 2 mmHg, with mild right ventricular dysfunction (Appendix A).

During the current pregnancy, first trimester combined screening indicated low risk for aneuploidy and preeclampsia. Serologies were negative except for positive IgG for rubella and CMV. All other obstetric assessments were normal.

At 8 weeks of gestation, she was admitted due to dyspnea and asthenia, with hospitalization extending until week 13. Chest radiography and echocardiography were performed to assess prosthetic valve function. Prosthetic valve thrombosis was diagnosed following the transition from warfarin to LMWH (enoxaparin 60 mg every 12 h).

Termination of pregnancy was considered; however, the patient opted to continue. A multidisciplinary team (Cardiology, Hemostasis, Cardiovascular Surgery) decided on conservative management given her clinical and echocardiographic stability. Enoxaparin was progressively increased to 100 mg every 12 h, combined with aspirin 100 mg. At week 12, therapy was switched to warfarin (4.5 mg/day) plus aspirin, with close monitoring including clinical assessment, coagulation parameters (INR 4–4.5), and echocardiography. Slow infusion fibrinolysis with alteplase was planned should symptoms recur.

During the initial hospitalization and throughout the pregnancy, weekly transthoracic echocardiograms were performed. Imaging showed progressive worsening of hemodynamic parameters, reaching an expiratory mean gradient of 8 mmHg and inspiratory gradient of 14 mmHg, with one leaflet fixed in an intermediate position. Improvement began around week 11 of gestation, stabilizing thereafter with a mean gradient of approximately 9–10 mmHg until delivery.

Serial fetal ultrasounds were performed, including detailed anatomical scans at 16 and 20 weeks and an advanced fetal echography at 24 weeks, with no malformations attributable to warfarin exposure identified.

The patient remained asymptomatic until week 31, when she was admitted for conversion to LMWH and delivery planning.

Pregnancy was concluded at 33 + 6 weeks by cesarean section under ischemic conditions, following administration of two doses of corticosteroids for fetal lung maturation.

Cesarean delivery was performed under general anesthesia, resulting in the birth of a male infant weighing 2600 g, with Apgar scores of 6 at 1 min and 8 at 5 min, an arterial pH of 7.19, and a venous pH of 7.21. The newborn was transferred to the Neonatology Unit for further evaluation. Postoperative maternal echocardiography showed no significant changes compared with previous studies.

During the postpartum period, anticoagulation with heparin and aspirin was resumed, followed by transition to warfarin prior to hospital discharge. Total hospitalization time was 29 days.

Four months postpartum, the patient underwent endoscopic replacement of the mechanical prosthesis with a 31 mm St. Jude Epic porcine bioprosthesis, with good subsequent functional outcomes. Intraoperative findings revealed the anterior leaflet fixed in an open position and the posterior leaflet in a semi closed position.

## 3. Discussion

Mechanical tricuspid valve thrombosis (MTVT) highlights one of the most severe and challenging cardiovascular complications that can occur during pregnancy. To our knowledge, no other cases have been described in the literature of a pregnant patient with a thrombosed mechanical heart valve who was able to progress to fetal viability.

It is well established that pregnancy induces a hypercoagulable state and marked hemodynamic changes, significantly increasing the risk of thromboembolism in patients with mechanical heart valves [9,14]. Thrombosis develops more frequently in right-sided valves, particularly in the tricuspid position [3,7,8], due to the physiological changes inherent to pregnancy, such as increased total blood volume, heart rate, and cardiac output, along with reduced systemic vascular resistance [1,5,9,13]. For this reason, pre-pregnancy counseling regarding the risks and benefits of anticoagulation regimens is essential [6,12,15,16].

According to the ROPAC III study, the probability of an uncomplicated pregnancy resulting in a live birth is approximately 54% [3,6]. This elevated maternal risk necessitates specialized management in tertiary care centers by a multidisciplinary team comprising specialists in obstetrics, cardiology, cardiac surgery, and hematology [1,2,4]. Throughout pregnancy, trimester-based echocardiographic evaluations should be performed to assess valve function and rule out thrombosis [6].

Prosthetic valve thrombosis should be suspected when changes in valvular gradients occur or when new symptoms arise, including heart failure, embolic events, syncope, or alterations in prosthetic clicks or murmurs. Diagnosis must be confirmed through transthoracic echocardiography (TTE), transesophageal echocardiography (TEE), computed tomography, or fluoroscopy [6,17].

Clinical guidelines recommend appropriate anticoagulation in patients with mechanical valves. In non-pregnant patients, the first line therapy is typically warfarin (vitamin K antagonists, VKAs) to achieve a therapeutic INR (3–4). However, because warfarin is classified as FDA category D, initial anticoagulation during pregnancy should be performed with unfractionated heparin (UFH) or low-molecular-weight heparin (LMWH) [1,5,7,8,9,10]. Mechanical valve thrombosis (MVT) frequently occurs during transitions from VKAs to heparin, particularly in the first trimester, making individualized anticoagulation strategies essential [5,10,11,12,13].

### 3.1. High Risk Associated with Mechanical Tricuspid Valves and Anticoagulation Management

The presence of a mechanical tricuspid valve (MTV) significantly increases both maternal and fetal risk. Tricuspid prostheses carry the highest risk of thrombosis among all valve positions (11.7-fold increase) [3,6,7], and therefore patients often require higher INR targets [7]. This predisposition to thrombosis is related to the lower flow velocity in the right heart and the typically larger size of tricuspid prostheses [18].

Acute management depends on the type of valve, degree of obstruction, maternal hemodynamic status, and gestational age. Because mechanical valve thrombosis is a potentially life threatening emergency, immediate consultation with a multidisciplinary team is essential [4,6].

#### 3.1.1. Anticoagulation with Warfarin (VKAs) vs. Heparins (LMWH/UFH) [1,2,4,14]

This approach may be considered in subacute settings, particularly in hemodynamically stable patients [3,6,7]. Warfarin carries the lowest risk of mechanical valve thrombosis (0–4%); however, its use during the first trimester—especially at doses > 5 mg/day—significantly increases the risk of fetal loss and teratogenicity compared with heparin, making heparins safer for the fetus [1,4,11,12,14]. If anticoagulation was adequate prior to the thrombotic event, intensification of VKA therapy or the addition of low dose aspirin may be considered [17].

Warfarin is recommended throughout pregnancy when doses ≤ 5 mg/day are sufficient, with INR monitoring every 1–2 weeks and switching to UFH at least two weeks before planned delivery (around week 36) [11]. UFH can then be discontinued 4–6 h before cesarean delivery. When LMWH is used, it should be replaced by UFH at least 36–48 h before delivery [3,6,15].

If higher doses of warfarin are required or the risk is unacceptable to the patient, switching to LMWH or UFH between weeks 6–12 is recommended, with strict monitoring of anti Xa levels (target 0.8–1.2 IU/mL, 4–6 h post dose). This strategy may also be considered in patients with lower thrombotic risk. However, the effectiveness of LMWH in preventing thrombosis remains debated, even with anti Xa monitoring (5.8–7.4% thrombosis with LMWH; 33% with UFH) [3,6,7,12,13,15].

The transition from VKAs to heparins is recognized as an extremely high risk period for the development of mechanical valve thrombosis [1,2,5]. Individualized risk–benefit assessment is essential [18].

#### 3.1.2. Thrombolytic Therapy

Thrombolysis may be considered particularly for right-sided thrombosis, in non-critical patients, when surgery is not feasible or carries prohibitive risk, or when anticoagulation strategies fail [3,6,7]. Low dose tissue plasminogen activator (tPA) (25 mg), administered in repeated slow (6 h) or ultra slow (25 h) infusions under TEE guidance, has shown success in pregnant women (68.2%), without evidence of increased complication rates. However, evidence remains limited, its use is controversial, and it is considered a relative contraindication during pregnancy due to a 20% risk of miscarriage [7,8,10,19,20].

#### 3.1.3. Cardiac Surgery

Surgery is indicated in critically unstable patients with acute thrombosis, severe obstruction or regurgitation, or large thrombosis (≥10 mm) complicated by embolism, particularly when medical therapy fails [3,6,8,13,15,20]. It carries high maternal–fetal risk. Although maternal mortality during cardiopulmonary bypass is comparable to that of non-pregnant patients, fetal mortality is high, reaching up to 33% [15].

If surgery is unavoidable, the optimal timing is between 13 and 28 weeks [15]. Performing surgery in the third trimester increases the risk of preterm birth and maternal complications. Conversely, when surgery is performed after cesareandelivery, particularly beyond 28 weeks, fetal survival improves without increasing maternal mortality.

This condition underscores the complexity of balancing maternal safety with fetal viability.

### 3.2. Management of Late Pregnancy in Women with Mechanical Tricuspid Valve Thrombosis

Late pregnancy management in women with mechanical tricuspid valve thrombosis represents a high risk scenario requiring meticulous planning and execution by a specialized team. The primary goal is to achieve a safe delivery while minimizing maternal thromboembolic and hemorrhagic risks associated with anticoagulation. An individualized plan regarding timing and mode of delivery is essential.

#### 3.2.1. Anticoagulation Transition

Switching from VKAs to UFH/LMWH should occur at least two weeks before delivery and no later than 36 weeks [1,11,12,15].

#### 3.2.2. Delivery Planning

Delivery is performed by cesarean section. This is a high risk moment due to the rapid shift in blood volume (*autotransfusion phenomenon*) following fetal and placental evacuation, which may destabilize patients with pre-existing right heart overload.

The “autotransfusion phenomenon” refers to the sudden return of approximately 500 mL of blood to the maternal circulation after delivery and placental expulsion. This increases central venous pressure and may precipitate heart failure, particularly in patients with elevated right-sided pressures associated with mechanical tricuspid valve thrombosis [16].

To mitigate this phenomenon, a highly specialized technique—cesarean section under ischemic conditions with subsequent hysterectomy—has been developed [16]. This technique occludes uterine circulation before fetal extraction, preventing the abrupt shift in uterine blood volume into the maternal circulation.

This approach is considered safe for the fetus, as the transient interruption of uterine arterial flow does not compromise fetal oxygenation due to rapid fetal extraction and the residual uteroplacental blood volume.

Ethical considerations must also be addressed, particularly the loss of fertility associated with this technique. Patients must be thoroughly counseled regarding the maternal morbidity and mortality risks of this approach compared with standard cesarean delivery, although it is typically reserved for conditions in which pregnancy is already contraindicated.

### 3.3. Surgical Technique: Cesarean Section Under Ischemic Conditions Followed by Hysterectomy [16]

The procedure must be performed in an operating room by a specialized multidisciplinary team including anesthesiologists, obstetricians, neonatologists, and cardiovascular surgeons. It may be performed under spinal anesthesia or general anesthesia, depending on surgical risk.

The patient is placed in the supine position, and a midline infra-supra umbilical laparotomy is performed. The vesicouterine fold is dissected, and the uterine vessels are identified.

Once vascular structures are clearly identified, the utero-ovarian pedicle and round ligament are clamped up to the uterine segment, ensuring closure of the broad ligament venous plexus. The uterine pedicle is then clamped perpendicular to the parametrium with the objective of inducing ischemic conditions while minimizing venous return.

After uterine vascular occlusion, the uterine segment is opened and the fetus delivered, with immediate clamping of the umbilical cord.

Without removing the placenta, a subtotal hysterectomy is performed without applying hemostatic sutures to the specimen, allowing the uterus to exsanguinate and thereby reducing the risk of autotransfusion. Finally, both round and utero-ovarian ligaments are ligated to ensure adequate hemostasis.

#### Immediate Postpartum Management

This period remains high risk for both hemorrhage and recurrence of mechanical valve thrombosis. Anticoagulation should be restarted within 24–48 h using prophylactic or intermediate dose LMWH [1], with transition to VKAs between postpartum days 5 and 7. In cases of hemorrhage, tranexamic acid 1 g IV is recommended after delivery.

## 4. Conclusions

The diagnosis of thrombosis in a mechanical tricuspid valve during pregnancy represents a clinical scenario of extremely high maternal and fetal risk. Management requires a multidisciplinary team to coordinate therapeutic decisions regarding anticoagulation, delivery planning, and postpartum care. It is essential to counsel patients on the risks of pregnancy in the presence of this underlying condition and to optimize their clinical status prior to conception.

This article presents the management of a rare and complex case in a tertiary care center and introduces the possibility of cesarean delivery under ischemic conditions as an alternative in situations of extreme risk.

## Figures and Tables

**Figure A1:**
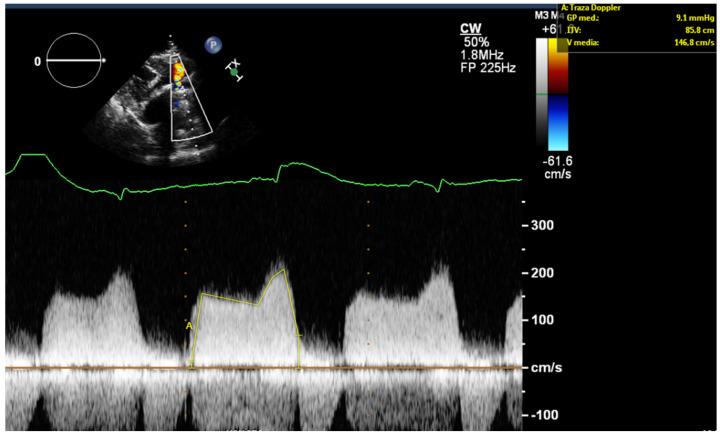
Elevated Trans—Tricuspid Gradients in the Setting of Prosthetic Valve Thrombosis.

## Data Availability

No new data were created or analyzed in this study. Data are not publicly available due to patient privacy and ethical restrictions.

## Abbreviations

The following abbreviations are used in this manuscript:

CMV	Cytomegalovirus
UFH	Unfractionated heparin
LMWH	Low-molecular-weight heparin
MHVs	Mechanical heart valves
MVT	Mechanical valve thrombosis
MTVT	Mechanical tricuspid valve thrombosis
TEE	Transesophageal echocardiography
TTE	Transthoracic echocardiography
VKAs	Vitamin K antagonist
